# Household Hints and Recipes

**Published:** 1876-10

**Authors:** 


### Household Hints & Recipes.

EXTRACT OF NEW-MOWN HAY.—A correspondent of the *Pharm. Jour. & Trans.* sends the following formula: Extract of tonquin, two pints; extracts of geranium, orange flower, rose, rose triple, and jasmine; of each one pint.

---

WATER-PROOFING GOODS.—Eight litres water are heated to 80° C., and a mixture of 300 grms. gelatine, and 600 grms. castor oil soap introduced, and then 300 grms. of gum lac. The fire is then withdrawn and 600 grms. of finely pulverized alum introduced, little by little, to insure complete solution. The solution thickens, giving a mixture which may be applied to fabrics with a fine brush, rendering them water-proof.

---

VEGETABLE LEATHER FROM SEA WEED.—Sheets of carded wadding are placed on hot polished metal plates, and coated with a concentrated decoction of sea weed, lichen, pearl moss, or other mucilaginous vegetation. The sheet is then dried quickly, thus giving to the surface applied to the metal plate a gloss like that of leather. Rolling and compressing between heated cylinders follows, and then a coating of boiled linseed oil is applied. Afterwards a thin coating of vegetable wax is given, and another rolling to soften the sheets finishes its preparation, when it is ready for bronzing or any other treatment.

---

TO FIX PENCIL DRAWINGS.—A correspondent of the *English Mechanic* gives the following directions for fixing pencil drawings: Lay the drawing on a sloping board, and pour boiling water gently over it; this will remove all superfluous particles of lead, and will bring some of the size in the paper to the surface; boil some isinglass or gum arabic in water to make a very thin size; pour it out on a flat dish to cool; run the drawing through the size, taking care that every part is well wetted; then lay it on a board to dry. The size should be so thin as to feel just a little sticky between the finger and thumb when cool. If too thick, it will be seen on the drawing after it is dry. I have tried many ways of fixing drawings, but have never found any to equal this. Another writer says: "The best solution to fix drawings is that made with gum tragacanth. It sizes the paper; it fixes the pencil drawings; it does not chip when wetted; it enables you to continue the drawing afterward if desired, and it is possible to color over it."

A GOOD KALSOMINE.—Take 4 lbs. Paris white, put in a pail, cover it with cold water, and let it stand over night; put into a tin kettle a handful of glue, cover with cold water; in the morning set the glue on the stove, and add enough warm water to make a quart, and stir until dissolved; add the glue to the Paris white, stir well, and pour in enough warm water to make a pail three-quarters full; then add blueing, a little at a time; stir well until it is very slightly bluish. Use a good brush; go over one place in the wall until thoroughly wet; if your brush dries quickly, add more warm water, as the mixture is too thick; the brush must be kept wet.—*Scientific American.*

---

CLEANING ENGRAVINGS.—Presuming these to be mounted, proceed in the following manner: Cut a stale loaf in half with a perfectly clean knife; pare the crust away from the edges. Now place the engravings on a perfectly flat table, and rubbing the surface with the fresh-cut bread, in circular sweeps, lightly but firmly performed, will remove all superficial markings. Now soak the prints for a short time in a dilute solution of hydrochloric acid, say 1 part of acid to 100 parts of water, and then remove them into a vessel containing a sufficient quantity of clear chloride of lime water to cover them. Leave them here until bleached to the desired point. Now remove, rinse well by allowing to stand an hour in a pan, in which a constant stream of water is allowed to flow, and finally dry off by spreading on clean cloths. Perhaps the sheets may require ironing between two sheets of clean paper.—*The Cabinetmaker.*

---

A CISTERN AND HOW TO BUILD IT.—Mr. Zimmerman thus writes to the *Evening Post:*

"For the benefit of your farmer readers, I will tell my method of making a rain-water cistern: The ground was loose gravel; I had it dug oval in shape, like a wash boiler, large enough to hold one hundred barrels. Then I bricked it up with common hard brick (arch brick will do), laid in water lime, with a cross-wall eight inches thick, also laid in water lime. This cross-wall I left one-third the distance on one side of the wall, and two-thirds on the other. It was firmly connected to the outside walls at each end, so that no leak could occur there; otherwise, it was laid up as a mason would lay up the wall of a house. This was arched over, leaving a 'man-hole' on each side of the dividing wall. The water entered on the large side; the pump drew it from the smaller. Not a drop of water could get into the smaller sections except by filtering through the cross-wall. This cistern has been in use nearly two years, and is a perfect success. Of course a waste-pipe is needed to carry off surplus water during heavy rains. The water is sweet, clear, and good to drink."

A CHEAP PAINT.—Many a farmer lives in an unpainted house which has become gray with age, surrounded by weather-beaten fences and unpainted sheds. The occupant of such a house cannot help appreciating the contrast between the cheerful aspect of his neighbor's well-painted buildings and fences and his own cheerless premises; but he sighs that he cannot afford the necessary expense, and contents himself as best he may with his unpleasant surroundings. Such an one will appreciate the value of the receipt which we give below, and which is said to almost equal the best white paint in appearance—though very cheap—and to be very durable. It was sent out by the U. S. Treasury Department. Every man who has an unpainted shed or other building on his premises should try it without fail.

"Slake half a bushel of unslaked lime with boiling water, keeping it covered during the process. Strain it, and add a peck of salt dissolved in warm water, three pounds of ground rice put in boiling water and boiled to a thin paste, one-half pound powdered Spanish Whiting, and a pound of clear glue dissolved in warm water; mix these well together, and let the mixture stand for several days. Keep the wash thus prepared in a kettle or portable furnace, and when used, put it on as hot as possible with either painters' or whitewash brushes."

PRESERVES.--*To Bottle Pineapples.*—Pluck off the heads and stalks, use a very sharp knife to pare away all the rind smoothly and without waste; with the point of a small knife pick or scoop out all the brown specks, cut the pine either in slices or finger-like pieces, and place them as close as possible in their bottles; fill up with twenty-six degrees syrup, cork and tie down. Time, twenty-five minutes ebullition.

Another way is to peal the pineapple, and scoop out the center right through to the extent of one inch in diameter; put the pine into a three-pound jar (supposing it to weigh about one pound and a half), fill the inside and all around the fruit with pounded sugar, cover the jar with a lid or paper, and place it in the screen, moderate the heat, and allow it to remain there until the sugar is dissolved into a syrup; then fill up the jar with more sugar, and as soon as this second lot has also become dissolved into a syrup, the jar may be covered down in the usual way and set aside in the cool. If, when peeling the pineapples, you at first remove the roughest portion of the exterior only, you may then pare off the rind a little thicker without fear of waste, as the rind can be placed in bottles and preserved according to either of the foregoing methods, and will become useful in making ices, creams, flavoring punch, etc.

*To Preserve Apples.*—Peel smoothly, and neatly scoop out the cores from the halves or quarters of apples; and, as they are turned out of hand, drop them into a white pan containing cold water acidulated with the juice of a couple of lemons and a small quantity of alum; next, let the apples be scalded with this acidulated water in a copper preserving pan over the fire without allowing the water to boil—it should barely simmer; and, as soon as you find that the apples are warmed through, let them be drained on a sieve, and run some cold water over them to refresh the apples; they are then to be closely placed in their bottles without jamming, filled up with twenty-four degrees syrup which has been slightly acidulated with citric acid and alum; cork, and tie down. Time, fifteen minutes gentle ebullition.

*To Bottle White Pears.*—When the pears are not large, they may be turned or peeled whole, you may leave the stalk on, merely scraping off its bark; but when large, they should be divided into halves or quarters, and, as they are turned out of hand, drop them into a pan containing cold water slightly acidulated with lemon juice and a pinch of bruised alum; parboil the pears in this water without allowing them to boil, and when about half-done, refresh the fruit in two separate cold waters, drain it on a sieve, and fill the bottles carefully and neatly without pressure; fill up with thirty-eight degrees syrup slightly acidulated with citric acid and alum; cork and tie down. Time, fifteen minutes ebullition.

*To Bottle Pink Pears.*—Proceed as indicated in the foregoing article, with this difference only; just enough prepared cochineal should be added when parboiling the pears, and also to the syrup in which they are preserved; care being taken to avoid charging the pears with any more color than will suffice to given them a delicate pink tinge.

*To Bottle Pears for Making Ices.*—Peel, core and cut up small, any fine-flavored ripe pears, into bottles, and as each bottle is so filled add twenty-two degrees syrup; cork, and tie down. Time twenty minutes ebullition.

*To Bottle Pears Whole.*—Small pears are best for *compotes* as well as for being either dried,

*glace*, or crystallized. Let these be prepared and bottled as directed for the white or pink pears; and when corked and tied down, they are to be steamed in the usual way. Time, fifteen minutes ebullition.

*To Bottle Large Plums.*—All large plums should be first pricked with a needle and dropped out of hand into a preserving pan containing twenty-two degrees syrup just off the fire; when all your fruit is in the syrup, set the pan (covered over to keep out the dust, &c.) either inside a hot screen or else upon a trivet over a smothered charcoal fire, until the syrup becomes quite hot; the plums must then be removed carefully with a large confectionery spoon into a white pan, and set aside until the next day, in order that they may become charged with sufficient syrup to give them substance; and, on the following day, use a spoon to fill the bottles with the plums so far prepared; give their syrup a boil up, and skim it, and when nearly cold fill up the bottles; cork, and tie down. Time, fifteen minutes ebullition.

*To Bottle Cherries.*—Let the cherries be picked from their stalks into the bottles, shaken down lightly without bruising, filled up with twenty-four degrees syrup; corked, and tried down. Time, ten minutes ebullition.—*The Confectioner.*

---

STAINS.—Queries in regard to the best methods for erasing various stains, are often sent by correspondents of *The Druggists' Circular*. The process recommended varies, of course, according to the kind of stain, and to the nature of the fabric.

We find in the *Apothek. Ztg. and Jour. de Phar d'Alsace-Lorraine* a table, concise as well as complete, giving directions for removing nearly all sorts of stains and spots from linen, cotton, woolen and silk fabrics. A reproduction of the article will, we believe, be interesting and useful to our readers.

The stains easiest to remove are those of SUGAR, GELATIN, BLOOD and ALBUMEN; a simple washing with water is all that is necessary for all kinds of fabrics.

GREASE SPOTS.

For *white linen or cotton goods*, use soap or weak lyes.

For *colored calicoes*, warm soap-suds.

For *woolens*, soap-suds or ammonia.

For *silks*, benzine, ether, ammonia, magnesia, chalk, yolk of egg with water.

PAINT, VARNISH, AND RESIN STAINS.

For *white or colored cotton or woolen* goods, oil of turpentine or benzine, followed by soap-suds.

For *silk*, benzine, ether, soap; hard rubbing is to be avoided.

STEARIN, SPERM CANDLES.

For all kinds, use 95 per cent. alcohol.

WINE AND FRUIT STAINS.

*White cotton or linen*, fumes of burning sulphur, warm chlorine water.

*Colored cottons or woolens*, wash with tepid soap-suds or ammonia.

*Silks*, the same, with very gentle rubbing.

ALIZARINE INK.

*White cottons and linens*, tartaric acid in solution; the older the stain, the more concentrated the solution should be.

*Colored cottons, woolens, and silks*, a weak solution of tartaric acid, if the color allows of its use.

RUST, NUTGALL INK.

*White cottons and linens*, warm solution of oxalic acid, dilute muriatic acid, followed by granulated tin.

*Colored cottons and woolens*, repeated washings with a solution of citric acid, if the color is fast.

*Silks*, do nothing; all attempts only make things worse.

LIME, LYE, ALKALIES.

*White cottons and linens*, wash with cold water.

*Colored goods and silks*, a weak solution of citric acid applied with the tip of the finger to the spot previously moistened with water.

ACIDS, VINEGAR, ORANGE JUICE, ETC.

*White cottons and linens*, wash with pure water, or warm chlorine water.

*Colored goods or silks*, ammonia, diluted according to the fineness of the tissue and the delicacy of the color.

TANNIN, WALNUT SHELLS.

*White cottons and linens*, javelle water, warm chlorine water, concentrated solution of tartaric acid.

*Colored goods or silks*, chlorine water, diluted according to the tissue and its color, each application to be followed by washing with water.

TAR, AXLE GREASE.

*White cottons and linens*, soap, oil of turpentine, and water, each applied in turns.

*Colored cottons and woolens*, first smear with lard, rub with soap and water, and let it stand a short time; then wash with oil of turpentine and turpentine and water, alternately.

*Silks*, the same, using benzine instead of turpentine, and drop the water from a certain height on the under side of the stain. Avoid rubbing.

